# Baicalein Protects H9c2 Cardiomyoblasts Against LPS-Induced Inflammatory Injury by Modulating the NF-κB/NLRP3 Inflammasome Pathway and Mitochondrial ROS

**DOI:** 10.5812/ijpr-169689

**Published:** 2026-04-28

**Authors:** Yi Lu, Zahra Zahid Piracha, Umar Saeed, Fangjun Luo

**Affiliations:** 1 Department of Rehabilitation, Sengong Hospital of Shaanxi Province, Xian, China; 2 Faculty of Rehabilitation and Allied Health Sciences, Riphah International University, Islamabad, Pakistan; 3 Széchenyi István University, Győr, Hungary; 4 Institute of Graduate Studies and Research, Cyprus International University, Nicosia, Cyprus; 5 Korea University College of Health Sciences, Korea University , Seoul, South Korea; 6 Department of Emergency, Hanzhong Hospital of Traditional Chinese Medicine, Hanzhong, China

**Keywords:** Baicalein, Lipopolysaccharide, H9c2 Cardiomyoblasts, NF-κB Signaling, NLRP3 Inflammasome, Caspase-1, IL-1β, IL-18, Oxidative Stress, Mitochondrial Membrane Potential, Reactive Oxygen Species, Sepsis-associated Cardiomyopathy, Cardiomyocyte Inflammation, Antioxidant Therapy

## Abstract

**Background:**

Sepsis-related cardiomyopathy is mainly induced by uncontrolled inflammation and mitochondrial oxidative damage triggered by endotoxins, especially those of Gram-negative bacteria. Lipopolysaccharide (LPS), an endotoxin, activates the Toll-like receptor 4/nuclear factor kappa B (TLR4/NF-κB) signaling pathway and the NOD-like receptor family pyrin domain containing 3 (NLRP3) inflammasome, which further activates caspase-1 to process the pro-inflammatory cytokines interleukin-1β (IL-1β) and interleukin-18 (IL-18). Chronic activation of this signaling pathway is responsible for mitochondrial damage and loss of membrane potential. Baicalein, a flavonoid derived from the herbal plant *Scutellaria baicalensis* is well recognized for its anti-inflammatory and antioxidant properties, although its full modulation of the NF-κB/NLRP3 mitochondrial signaling pathway is not thoroughly understood.

**Objectives:**

In this study, we assessed whether baicalein has a protective effect on H9c2 cardiomyoblasts against inflammatory and mitochondrial injury induced by LPS through regulation of inflammatory pathways involving NF-κB, the inflammasome, caspase-1 activity, cytokine expression, and oxidation.

**Methods:**

H9c2 cells were pre-exposed to different baicalein concentrations (5 - 20 µM) and, after 1 hour, exposed to 1 µg/mL of LPS. Analysis included cell viability measurements by MTT assay, lactate dehydrogenase (LDH) release, and microscopic evaluation by phase-contrast microscopy. Expression levels of premature Nlrp3, Il1B, Il18, and Casp1 (0 - 4 h) were analyzed by real-time polymerase chain reaction (PCR). Protein levels of inflammasome and NF-κB pathway proteins were evaluated by immunoblot analysis. Activity of caspase-1 and cytokine secretion of IL-1β and IL-18 were evaluated by colorimetry and enzyme-linked immunosorbent assay (ELISA), respectively. Mitochondrial membrane potential (ΔΨm) was evaluated by staining with JC1 dye, and reactive oxygen species (ROS) levels were evaluated by fluorescence with DCFH2DA; N-acetylcysteine (NAC) was used as an antioxidant positive control.

**Results:**

LPS strongly elevated the transcription of Nlrp3 and Il1b at 2 hours, in addition to enhancing protein expression of NLRP3, activity of caspase-1, and secretion of IL-1β and IL-18. Underlying these pro-inflammatory responses were mitochondrial depolarization, augmented ROS production, diminished cell survival, and cytotoxicity. Prior administration of baicalein mitigated NF-κB activation, diminished priming of the inflammasome response at both transcriptional and protein levels, and decreased caspase-1 activity and secretion of the cytokines. Moreover, baicalein maintained mitochondrial membrane potential and diminished ROS within the cells, comparable to NAC, and improved cellular viability and morphology

**Conclusions:**

Baicalein confers substantial protection against LPS-induced inflammatory and oxidative damage in H9c2 cardiomyoblasts by concurrently suppressing NF-κB activation, mitigating NLRP3 inflammasome signaling, curbing caspase-1–driven cytokine maturation, and stabilizing mitochondrial function through reduction of ROS. These findings identify baicalein as a promising candidate for targeting the ROS-inflammasome axis in sepsis-associated cardiac dysfunction and related inflammatory cardiac disorders.

## 1. Background

Inflammation-induced cardiac dysfunction lies at the heart of several cardiovascular diseases, including sepsis-associated cardiomyopathy, ischemia–reperfusion injury, and metabolic heart disorders, and continues to account for significant global morbidity and mortality (1-5). Among the methods used to simulate septic inflammation in myocardial cells, lipopolysaccharide (LPS), a component of the gram-negative bacterial cell membrane, is well recognized for its role in the activation of Toll-like receptor 4 (TLR4) in cardiomyocytes. Activation of TLR4 triggers activation of the MyD88/IKK/NF-κB pathway, resulting in exaggerated activation of the inflammatory response (6). Overactivation of the NF-κB signaling pathway stimulates the transcription of major pro-inflammatory genes and inflammasome components, which underlies the pathogenesis of cardiac muscle inflammation (7, 8).

One of the key components in this cascade is the NLRP3 inflammasome, a cytosolic multiprotein complex that is triggered by several stimuli, including mitochondrial reactive oxygen species (ROS), ionic changes, and cellular damage (9, 10). Upon activation, NLRP3 binds to ASC and pro-caspase-1, initiating the enzymatic processing of pro-interleukin-1β (IL-1β) and pro-interleukin-18 (IL-18) into their active forms (11). The latter mediates the promotion of inflammatory responses and contributes to decreased cardiac function, thus making NLRP3 a fundamental target in sepsis-induced cardiac damage (11-13).

In order to explore the underlying mechanisms of LPS-induced cardiomyocyte injury at the molecular level and assess potential protective strategies, H9c2, a rat cardiomyoblast cell line, is widely used. H9c2 cells are derived from embryonic ventricular cardiac cells and preserve their major characteristics, including a high mitochondrial density, oxidative metabolism, calcium responsiveness, and gene expression of Toll-like receptor 4 (TLR4) and the NLRP3 inflammasome (14). These characteristics make H9c2 cells an excellent and reliable experimental model to mimic LPS-induced NF-κB activation, NLRP3 inflammation, mitochondrial depolarization, and ROS-induced injury in vitro (15). Since oxidative stress is known to be inextricably connected with LPS-induced myocardial injury, ROS overproduction and mitochondrial instability offer a major intersection point between LPS-induced inflammation and the injury process in cardiomyocytes (16-18)

Baicalein, a naturally occurring flavonoid derived from *Scutellaria baicalensis*, has recently been recognized for its potent antioxidative, anti-inflammatory, and mitochondria-protective activities (19-21). However, previous studies have clearly demonstrated that baicalein possesses the capacity to inhibit NF-κB signaling, decrease ROS accumulation, and regulate inflammasome activity, although these properties have mostly been reported in isolation or in non-cardiac systems (22, 23). It is important to note that the interrelationship among the ability of baicalein to prime the transcription of inflammasome genes, activate caspase-1, process cytokines, maintain mitochondrial integrity, and regulate ROS production in the context of LPS-induced inflammation in the heart remains poorly elucidated. Indeed, the temporal regulation of the expression of genes encoded within the inflammasome, specifically the immediate transcriptional burst elicited after LPS exposure, has also remained poorly explored in cardiomyoblasts exposed to baicalein.

## 2. Objectives

This current study, thus, aimed to fully investigate the protective mechanisms of baicalein at different biological levels of LPS-mediated damage in H9c2 cardiomyoblast cells. Based on the analysis of cell viability, LDH release, gene expression, protein expression, activation of caspase-1, release of cytokines, mitochondrial membrane potential, and cellular ROS, the study provides in-depth insight into the effects of baicalein and its ability to regulate the NF-κB/NLRP3 pathway. With the earlier time-course analysis (from 0 - 4 hours) of inflammasome priming and the use of the antioxidant substance N-acetylcysteine (NAC), this study tries to confirm the specific underlying protective ability of baicalein that is more dependent on the regulation of ROS, NF-κB, and the inflammasome, and thus provides valuable mechanistic information that can be applied both in the context of inflammatory responses of the cardiovascular system in sepsis and in various oxidative pathologies of the heart.

## 3. Methods

### 3.1. Cell Culture

H9c2 rat cardiomyoblasts (ATCC^®^ CRL-1446™) were cultured in high-glucose Dulbecco’s Modified Eagle Medium (DMEM; Gibco, Cat. No. 11965-092) supplemented with 10% fetal bovine serum (FBS; Gibco, Cat. No. 16000-044), 1% penicillin-streptomycin (Gibco, Cat. No. 15140-122), and maintained at 37°C in a humidified 5% CO₂ incubator. Cells were seeded at 70–80% confluence and used between passages 4 - 15 for all experiments (14).

### 3.2. Reagents

- Lipopolysaccharide (LPS, *E. coli* O111:B4): Sigma-Aldrich, Cat. No. L2630

- Baicalein (≥ 98% purity): Sigma-Aldrich, Cat. No. 465119

- N-acetyl-L-cysteine (NAC): Sigma-Aldrich, Cat. No. A9165

- MTT reagent (3-[4,5-dimethylthiazol-2-yl]-2,5-diphenyltetrazolium bromide): Sigma-Aldrich, Cat. No. M2128

- LDH Cytotoxicity Detection Kit: Takara Bio, Cat. No. MK401

- JC-1 Mitochondrial Membrane Potential Assay Kit: Abcam, Cat. No. ab113850

- DCFH-DA ROS Detection Probe: Sigma-Aldrich, Cat. No. D6883

- TRIzol™ Reagent: Invitrogen, Cat. No. 15596026

- High-Capacity cDNA Reverse Transcription Kit: Applied Biosystems, Cat. No. 4368814

- SYBR™ Green PCR Master Mix: Applied Biosystems, Cat. No. 4367659

- Enzyme-linked immunosorbent assay (ELISA) Kits:

Rat IL-1β ELISA: Abcam, Cat. No. ab255730

### 3.3. Baicalein Pretreatment and LPS Stimulation

Cells were pretreated with baicalein (5, 10, or 20 µM; 1 h) diluted in complete medium. Lipopolysaccharide (1 µg/mL) was added for the indicated durations (0 - 24 h). Baicalein alone (10 µM) served as a non-toxic control. For antioxidant comparison, N-acetyl-L-cysteine (NAC) (5 mM; 1 h pretreatment) was used.

### 3.4. MTT Cell Viability Assay

Cell viability was assessed using the MTT assay. H9c2 cells were seeded in 96-well plates (1 × 10⁴ cells/well). After treatments, 10 µL of MTT solution (5 mg/mL) was added and incubated for 4 h at 37°C. The formazan crystals were dissolved in 100 µL dimethyl sulfoxide (DMSO), and absorbance was measured at 570 nm using a microplate reader (BioTek Synergy HTX). A separate calibration curve (1 - 8 × 10³ cells/well) was generated to confirm assay linearity (R² = 0.984) (24).

### 3.5. Lactate Dehydrogenase Release Assay

Membrane integrity was evaluated using the lactate dehydrogenase (LDH) Cytotoxicity Detection Kit following the manufacturer’s guidelines (25). Absorbance was recorded at 490 nm. LDH release (%) was calculated as:


$$\text{LDH Release}=\frac{\text{Sample LDH Activity}}{\text{Total Lysis Control}}\times 100$$

### 3.6. RNA Extraction, cDNA Synthesis, and Quantitative Real-time PCR

Total RNA was isolated using TRIzol™ reagent according to the manufacturer’s instructions. cDNA was synthesized from 1 µg RNA using the High-Capacity cDNA kit. Quantitative real-time PCR (qPCR) was performed on a QuantStudio™ 5 System (Applied Biosystems) using SYBR™ Green Master Mix. Reactions were performed under standard cycling conditions. Relative expression was quantified using the 2^⁻ΔΔCt ^method with Gapdh as the housekeeping control. For mechanistic analysis, ΔCt values at the 2 h peak (identified from time-course experiments) were statistically evaluated. Primer sequences for Nlrp3, Il1b, Il18, Casp1, and Gapdh are provided in Supplementary Table S1.

### 3.7. Protein Extraction and Western Blot Analysis

Cells were lysed in RIPA buffer (Thermo Fisher, Cat. No. 89900) supplemented with protease and phosphatase inhibitors (Sigma, Cat. No. P5726). Protein concentration was measured using the BCA assay (Thermo Fisher, Cat. No. 23225) (26). Twenty-five micrograms of protein per sample were resolved on 10 - 12% SDS-PAGE gels and transferred onto PVDF membranes (Millipore, Cat. No. IPVH00010). Membranes were blocked in 5% bovine serum albumin (BSA) (Sigma, Cat. No. A9647) and incubated overnight at 4°C with primary antibodies as shown in Table 1. 

**Table 1.** List of Primary Antibodies

Target	Supplier	Catalog No.
**NLRP3**	Cell Signaling Technology (CST; Danvers, MA, USA)	15101
**Caspase-1 (p20)**	AdipoGen Life Sciences (San Diego, CA, USA)	AG-20B-0042
**IL-1β (pro/p17)**	Cell Signaling Technology (CST; Danvers, MA, USA)	12703
**Phospho-NF-κB p65 (Ser536)**	Cell Signaling Technology (CST; Danvers, MA, USA)	3033
**NF-κB p65 (total)**	Cell Signaling Technology (CST; Danvers, MA, USA)	8242
**IκBα**	Cell Signaling Technology (CST; Danvers, MA, USA)	4812
**GAPDH**	Cell Signaling Technology (CST; Danvers, MA, USA)	5174

Membranes were incubated with horseradish peroxidase (HRP)-conjugated secondary antibodies (CST, Cat. No. 7074/7076) and visualized using ECL substrate (Thermo, Cat. No. 32106). Densitometry was performed using ImageJ v1.53.

### 3.8. Caspase-1 Activity Assay

Caspase-1 enzymatic activity was quantified using the Caspase-1 Colorimetric Assay Kit (Abcam, Cat. No. ab39412) (20). Cell lysates (200 µg protein) were incubated with YVAD-pNA substrate at 37°C for 2 h, and absorbance was measured at 405 nm.

### 3.9. Enzyme-Linked Immunosorbent Assay

Supernatants collected after 24 h of LPS stimulation were assayed for IL-1β and IL-18 using the ELISA kits listed above. Standards and samples were run in duplicate, and absorbance was measured at 450 nm.

### 3.10. Mitochondrial Membrane Potential (ΔΨm)

JC-1 dye staining was performed using ab113850. After treatment, cells were incubated with JC-1 working solution (10 µg/mL) for 20 min at 37°C. Red (J-aggregates) and green (monomers) fluorescence were imaged using a confocal microscope (Leica TCS SP8) with a 60× oil objective. ΔΨm was expressed as the red/green fluorescence ratio (27).

### 3.11. Intracellular ROS Measurement (DCFH-DA)

Cells were incubated with 10 µM DCFH-DA for 30 min at 37°C, washed twice with phosphate-buffered saline (PBS), and imaged by confocal microscopy (Leica TCS SP8, 40×). Quantitative fluorescence intensity was measured using ImageJ and normalized to cell number (28, 29).

### 3.12. Phase-Contrast Morphology

Cell morphology was visualized using an inverted phase-contrast microscope (Olympus CKX53, 20× objective). Images were collected before and after treatments to assess rounding, shrinkage, and detachment.

### 3.13. Statistical Analysis

Data are presented as mean ± standard deviation (SD) (n ≥ 3 independent experiments). Statistical analyses were performed using GraphPad Prism 9.0. Two-way analysis of variance (ANOVA) with Sidak post hoc test was used for time-course and dose–response qPCR. One-way ANOVA with Tukey post hoc test was applied for MTT, LDH, ELISA, ROS, JC-1, and caspase-1 assays. A P-value < 0.05 was considered statistically significant.

## 4. Results

### 4.1. Baicalein Protects H9c2 Cardiomyoblasts from LPS-Induced Cytotoxicity and Preserves Cellular Morphology

**Figure 1.**
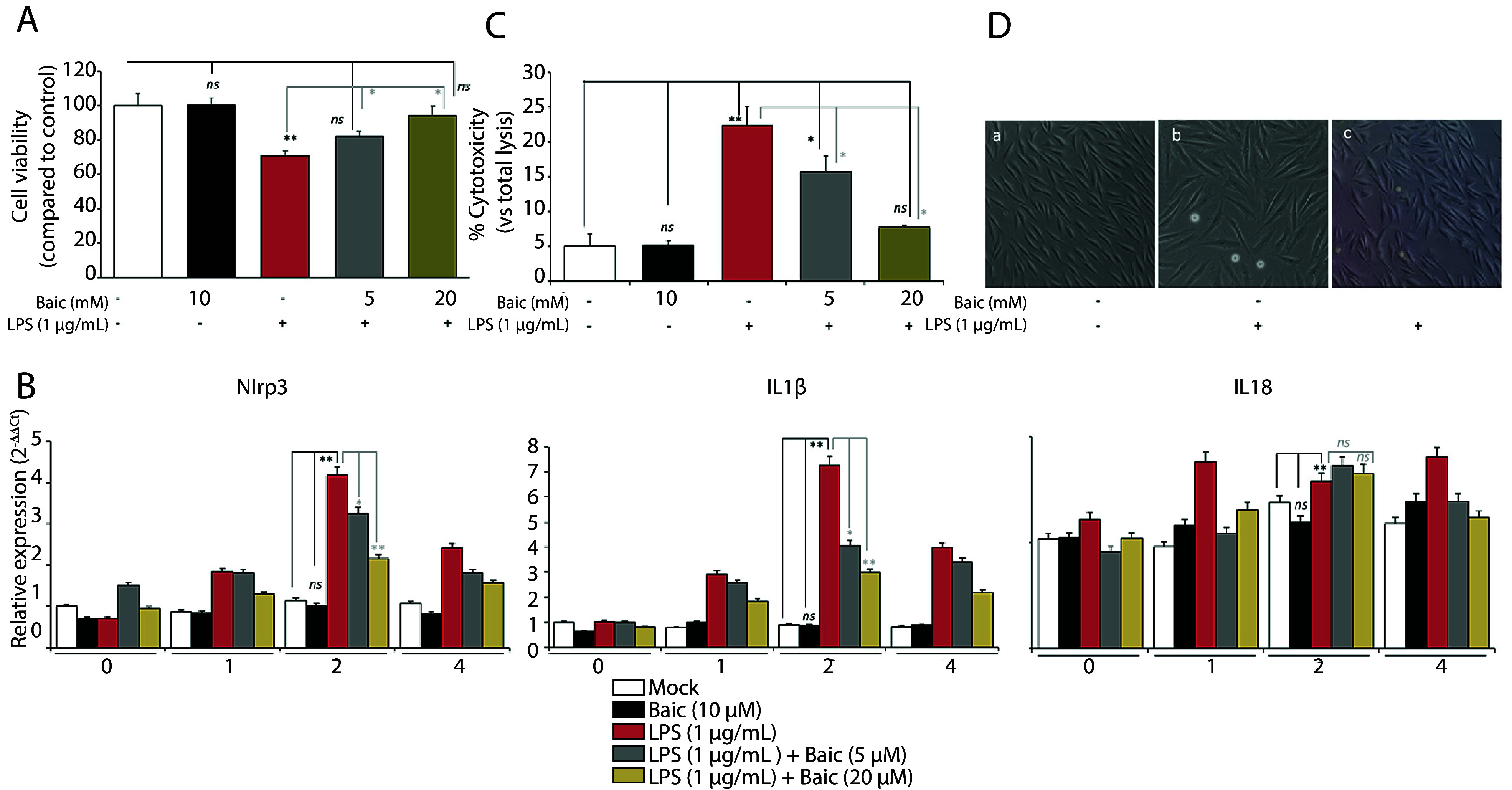
Baicalein preserves cell viability and morphology in lipopolysaccharide (LPS)-challenged H9c2 cardiomyoblasts. A, MTT cell viability assay. H9c2 cells were seeded at 1 × 10⁴ cells/well in 96-well plates and pretreated with baicalein (5 - 20 µM; 1 h), followed by LPS (1 µg/mL; 24 h). MTT reagent (5 mg/mL, Sigma M2128) was added for 4 h, formazan crystals were dissolved in DMSO, and absorbance was recorded at 570 nm (BioTek Synergy HTX). A calibration curve (1 – 8 × 10³ cells/well) was generated to validate linearity. Bars show mean ± SD. B, Inflammasome transcript kinetics (0 - 4 h). Total RNA was extracted using TRIzol™ (Invitrogen), converted to cDNA using the High-Capacity kit, and qPCR was performed with SYBR™ Green on a QuantStudio™ 5. Nlrp3, Il1b, and Il18 expression was evaluated from 0 - 4 h after LPS exposure to identify the mechanistic transcriptional peak. Relative abundance was calculated via 2⁻ΔΔCt using GAPDH as the reference. C, LDH release assay for membrane integrity. Supernatants were collected after 24 h of LPS stimulation, and LDH activity was quantified using the Takara MK401 kit. Absorbance at 490 nm was compared with the maximum lysis control to compute LDH release (%). D, Phase-contrast morphology. H9c2 cells were imaged on an Olympus CKX53 inverted microscope (20× objective) to assess rounding, shrinkage, and detachment following LPS exposure, and morphological preservation following baicalein pretreatment.

To establish a reliable model of LPS-induced inflammatory injury in H9c2 cardiomyoblasts and to determine a safe, effective concentration range of baicalein for subsequent mechanistic studies, the MTT assay was first employed to evaluate the effects of LPS (1 µg/mL, 24 h) and baicalein pretreatment (5 - 20 µM, 1 h) on cell viability (Figure 1A). Exposure to LPS alone reduced metabolic activity to approximately 70% of control values (P < 0.01), indicating moderate cytotoxic stress. Baicalein pretreatment exerted a clear concentration-dependent protective effect, restoring viability to 85–95% of control without detectable toxicity at any tested dose. The corresponding calibration curve for the MTT assay demonstrated linearity (R² = 0.984), confirming the reliability of the absorbance-to-cell-number relationship.

To characterize early transcriptional dynamics of inflammasome-related genes, quantitative real-time PCR was performed for Nlrp3, Il1b, and Il18 mRNA from 0 to 4 h following LPS stimulation (Figure 1B). All three transcripts displayed rapid induction kinetics, peaking at 2 h and declining thereafter. At this peak, LPS significantly upregulated Nlrp3 (~4-fold) and Il1b (~7-fold) vs control (P < 0.001), whereas Il18 showed a modest ~2-fold increase (P < 0.001 vs control). Baicalein alone (10 µM) had no effect on basal transcript levels (n.s. vs control). Baicalein pretreatment attenuated LPS-induced Nlrp3 and Il1b expression in a dose-dependent manner—LPS + Baic-5 µM vs LPS, P < 0.01; LPS + Baic-20 µM vs LPS, P < 0.01 — reducing mRNA abundance by roughly 40 – 50% at the higher dose. In contrast, Il18 mRNA remained unchanged by either concentration (LPS + Baic-5 µM vs LPS, n.s.; LPS + Baic-20 µM vs LPS, n.s.), consistent with its post-transcriptional control via caspase-1–mediated processing. These data confirm that LPS rapidly primes the NLRP3 inflammasome, while baicalein selectively blunts NF-κB-driven Nlrp3 and Il1b upregulation without affecting the more constitutive Il18 gene. Statistical analyses were performed on ΔCt values using two-way ANOVA with Sidak post hoc testing at the 2 h peak, whereas the full 0 - 4 h kinetics are shown descriptively to illustrate temporal behavior.

LDH release assays were then performed to determine whether baicalein’s anti-inflammatory actions coincided with membrane protection (Figure 1C). LPS exposure markedly increased LDH release to 22.27 ± 2.74% of total lysis vs 5.10 ± 0.63% in controls (P < 0.001), confirming substantial membrane damage. Baicalein alone (10 µM) was non-toxic (5.06 ± 1.67%, n.s. vs control). Co-treatment with baicalein significantly and dose-dependently reduced LPS-induced LDH release—15.62 ± 2.36% at 5 µM (P < 0.01 vs LPS; P < 0.01 vs control) and 7.67 ± 0.33% at 20 µM (P < 0.001 vs LPS; n.s. vs control) — indicating partial and full cytoprotection, respectively. These results demonstrate that baicalein effectively mitigates LPS-induced cytotoxicity in a concentration-dependent manner while maintaining baseline cell viability. Phase-contrast microscopy further corroborated these biochemical observations (Figure 1D). 

Control cells exhibited elongated, spindle-shaped morphology with uniform confluence. LPS-treated cells showed pronounced rounding, shrinkage, and detachment — hallmarks of inflammatory injury. Baicalein pretreatment (20 µM) visibly preserved cellular architecture, maintaining adherence and minimizing floating debris, indicative of partial restoration of normal morphology and structural integrity. Together, these results confirm that baicalein is well tolerated in H9c2 cells, prevents LPS-induced loss of viability, and attenuates early inflammasome gene activation. The morphological preservation and reduced LDH release further underscore its cytoprotective and anti-inflammatory potential in inflammation-linked cardiac injury.

### 4.2. Baicalein Suppresses LPS-Induced Inflammasome Activation and Downstream Caspase-1 Signaling

**Figure 2.**
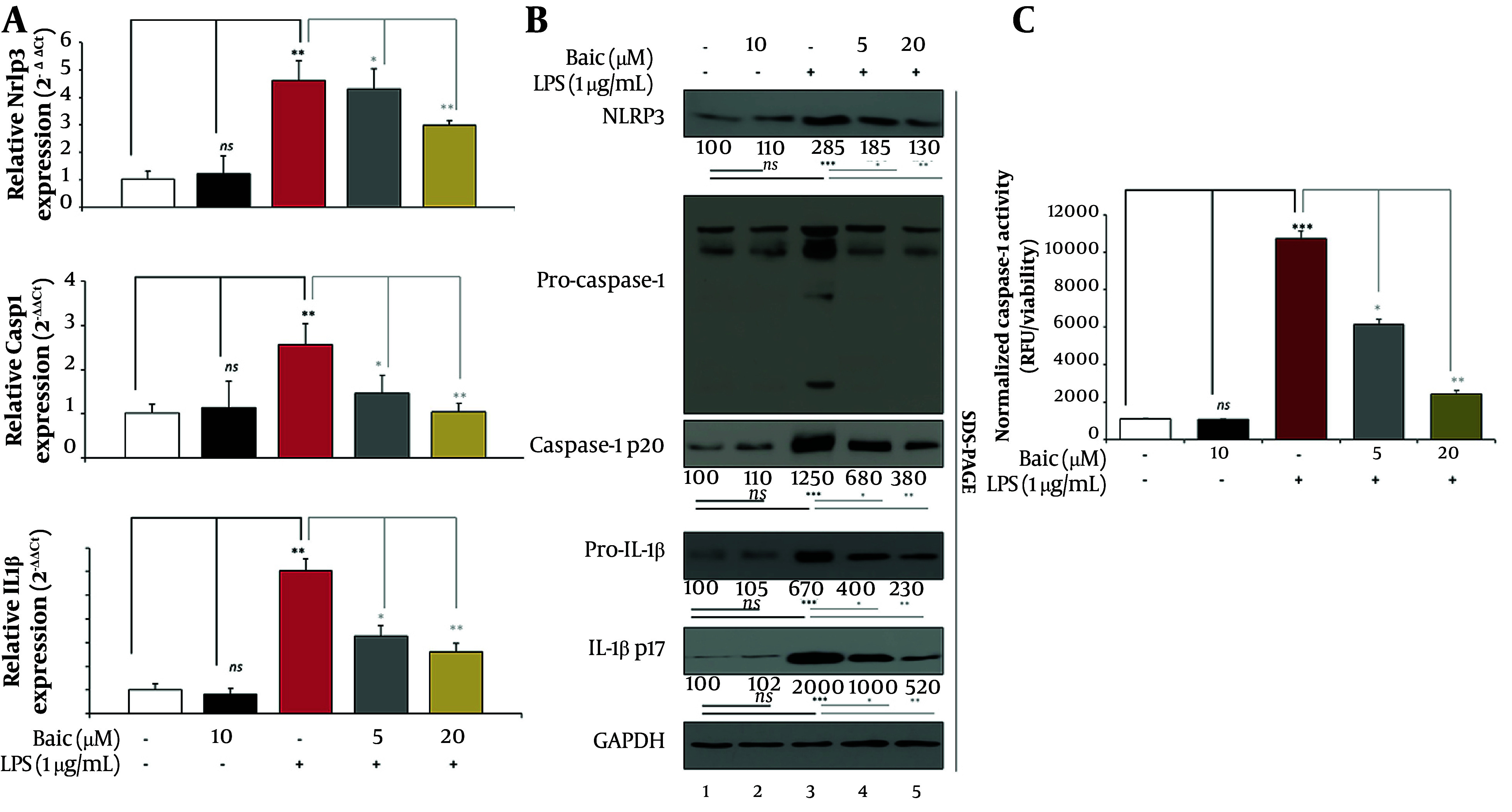
Baicalein modulates inflammasome signaling at transcriptional, protein, and enzymatic levels. A, qPCR analysis of Nlrp3, Casp1, and Il1b expression. Cells pretreated with baicalein (5 - 20 µM, 1 h) were stimulated with LPS for 2 h (the transcriptional peak defined in Figure 1). RNA isolation, cDNA synthesis, and qPCR conditions were identical to Figure 1B. B, Western Blot analysis of inflammasome-related proteins. Protein lysates were prepared in RIPA buffer containing protease/phosphatase inhibitors. Equal protein amounts (25 µg/lane) were resolved by SDS-PAGE and immunoblotted for NLRP3, caspase-1 p20, pro-IL-1β/IL-1β p17, and GAPDH. HRP-conjugated secondary antibodies and ECL substrate were used for detection. Representative blots and densitometry summaries are shown. C, Caspase-1 activity assay. Caspase-1 enzymatic activity was quantified using the Abcam ab39412 colorimetric kit. Cell lysates (200 µg protein) were incubated with YVAD-pNA substrate at 37°C for 2 h, and absorbance was measured at 405 nm.

Building on the time-course data from Figure 1B, which identified 2 h post-LPS as the transcriptional peak, we next analyzed key inflammasome-related genes to evaluate baicalein’s mechanistic impact on the NLRP3 axis (Figure 2A). 

Quantitative real-time PCR revealed that LPS stimulation markedly upregulated Nlrp3, Casp1, and Il1b mRNA (all P < 0.001 vs Control), confirming coordinated transcriptional activation of the priming and effector components. Baicalein pretreatment (5 - 20 µM) significantly suppressed these LPS-induced transcripts in a dose-dependent manner — LPS + Baic-5 µM vs LPS, P < 0.01; LPS + Baic-20 µM vs LPS, P < 0.001—while baicalein alone (10 µM) had no effect on basal expression (n.s. vs Control). These findings indicate that baicalein effectively inhibits transcriptional priming of the NLRP3 axis at the mechanistic peak identified in Figure 1B. Western blotting was used to assess inflammasome activation at the protein level (Figure 2B). Relative to control (set to 100%), LPS markedly increased NLRP3 (~285%), induced a ~12.5-fold rise in caspase-1 p20, and robustly elevated mature IL-1β p17 (~2000%) (all P < 0.0001 vs Control), confirming inflammasome activation. Baicalein pretreatment significantly reduced these elevations in a concentration-dependent manner: at 5 µM, NLRP3, p20, and p17 fell to ~100%, ~570%, and ~1000% of control, respectively (P < 0.01 vs LPS for each). Baicalein 20 µM further decreased all three readouts (P ≤ 0.01 - 0.001 vs LPS), trending toward baseline without altering basal protein levels when given alone (10 µM; n.s. vs Control). Pro-caspase-1 (~45 kDa) remained relatively stable across groups, consistent with post-translational activation rather than new synthesis. These data show that baicalein suppresses LPS-induced inflammasome activation by limiting NLRP3 accumulation and caspase-1–dependent IL-1β maturation.

Consistent with the transcriptional and protein data, baicalein also reduced inflammasome functional output, as shown by caspase-1 enzymatic activity (Figure 2C). LPS stimulation caused a dramatic activation, increasing activity to approximately 970% of control (P < 0.0001 vs Control). Baicalein pretreatment significantly attenuated this activation in a concentration-dependent manner: LPS + Baic-Low (5 µM) reduced activity to ~556% of control (P < 0.01 vs LPS), whereas LPS + Baic-High (20 µM) further suppressed it to ~219% of control (P < 0.001 vs LPS). Baicalein alone (10 µM) had no effect on basal caspase-1 activity (n.s. vs Control). These findings confirm that baicalein not only limits inflammasome priming and protein cleavage but also restrains the downstream enzymatic function of caspase-1 within the activated NLRP3 inflammasome complex. Together with the cytoprotective outcomes shown in Figure 1, these results establish that baicalein suppresses LPS-triggered inflammasome activation at multiple levels—transcriptional priming, protein maturation, and caspase-1 activation—thereby mitigating the inflammatory cascade responsible for cardiac cellular injury.

### 4.3. Functional Output and NF-κB Priming

**Figure 3.**
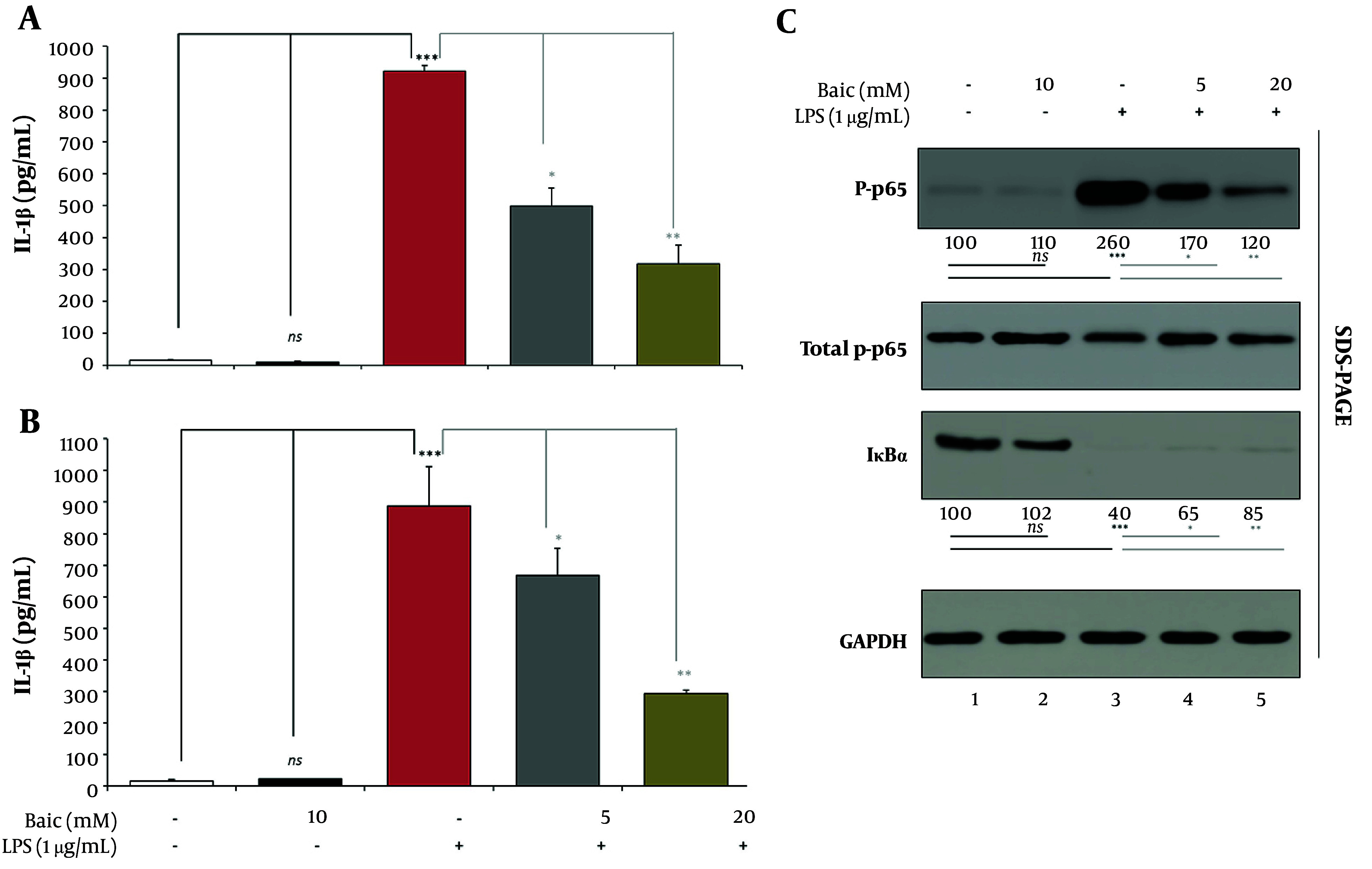
Baicalein reduces IL-1β and IL-18 secretion and limits NF-κB activation in H9c2 cells A and B, ELISA quantification of IL-1β and IL-18 secretion. Supernatants collected after 24 h of lipopolysaccharide (LPS) stimulation were analyzed using rat IL-1β (ab255730) and IL-18 (ab213909) ELISA kits (Abcam). Plates were read at 450 nm. C, NF-κB pathway Western blotting. Total and phosphorylated p65 (Ser536) and IκBα were evaluated by Western blot using primary antibodies (CST) and quantified by densitometry. Band intensities were normalized to total p65 for phosphorylated p65 and GAPDH for IκBα.

To assess the functional outcome of inflammasome modulation, IL-1β secretion was quantified by ELISA after 24 h of LPS stimulation (Figure 3A). LPS treatment caused a dramatic rise in IL-1β release (922.5 ± 17.1 pg/mL) compared with control cells (15.4 ± 3.1 pg/mL, P < 0.0001), confirming robust inflammasome activation. Baicalein alone (10 µM) had no effect on basal cytokine secretion (10.2 ± 2.7 pg/mL; n.s. vs Control). Co-treatment with baicalein markedly suppressed LPS-induced IL-1β release in a concentration-dependent manner: LPS + Baic-Low (5 µM) reduced secretion to 498.4 ± 56.2 pg/mL (P < 0.01 vs LPS), while LPS + Baic-High (20 µM) further decreased levels to 318.1 ± 57.3 pg/mL (P < 0.001 vs LPS).

These results demonstrate that baicalein effectively inhibits LPS-driven IL-1β secretion, reinforcing its suppressive action on inflammasome activation and downstream cytokine maturation. Parallel ELISA quantification of IL-18 secretion revealed a pattern broadly similar to that of IL-1β but with a smaller dynamic range (Figure 3B). 

LPS stimulation produced a pronounced rise in IL-18 release (887.8 ± 125.4 pg/mL) compared with control cells (22.7 ± 0.6 pg/mL; P < 0.0001), confirming activation of caspase-1–dependent cytokine processing. Baicalein alone (10 µM) had no effect on basal secretion (17.1 ± 3.7 pg/mL; n.s. vs Control). Co-treatment significantly and dose-dependently suppressed IL-18 release: LPS + Baic-Low (5 µM) reduced secretion to 667.4 ± 86.4 pg/mL (P < 0.05 vs LPS), whereas LPS + Baic-High (20 µM) further decreased levels to 294 ± 11.7 pg/mL (P < 0.001 vs LPS). Importantly, this post-transcriptional suppression aligns with the stable Il18 mRNA expression observed earlier at 2 h (Figure 1B), underscoring that IL-18 regulation occurs primarily through caspase-1–mediated cleavage and secretion rather than NF-κB–driven gene induction. Thus, the discrepancy between Il18 transcript and protein levels reflects distinct regulatory checkpoints — transcriptional priming (minimal) versus proteolytic maturation (robust) — both effectively restrained by baicalein. These data confirm that baicalein dampens the functional output of the NLRP3 inflammasome by limiting the release of mature IL-18, in parallel with reduced IL-1β secretion and caspase-1 activity.

To evaluate whether baicalein suppresses inflammasome priming at the transcriptional level, NF-κB activation was analyzed by Western blotting for phosphorylated p65 (p-p65), total p65, and IκBα (Figure 3C). LPS stimulation markedly enhanced p-p65 phosphorylation to approximately 260% ± 18% of control (P < 0.0001) while promoting substantial IκBα degradation to ~40% ± 6% of control (P < 0.0001), confirming canonical NF-κB activation. Baicalein pretreatment attenuated these changes in a clear concentration-dependent fashion: at 5 µM, the p-p65/p65 ratio declined to about 65% of the LPS level (P < 0.01 vs LPS), and at 20 µM, it fell further to 45% (P < 0.001 vs LPS). Correspondingly, IκBα protein abundance was preserved at ~65–85% of control, indicating reduced degradation and upstream blockade of NF-κB signaling. Together, these data demonstrate that baicalein inhibits both the priming (NF-κB) and effector (caspase-1/IL-1β/IL-18) phases of inflammasome activation in H9c2 cardiomyoblasts.

### 4.4. Baicalein Preserves Mitochondrial Membrane Potential (ΔΨm) and Morphology

**Figure 4.**
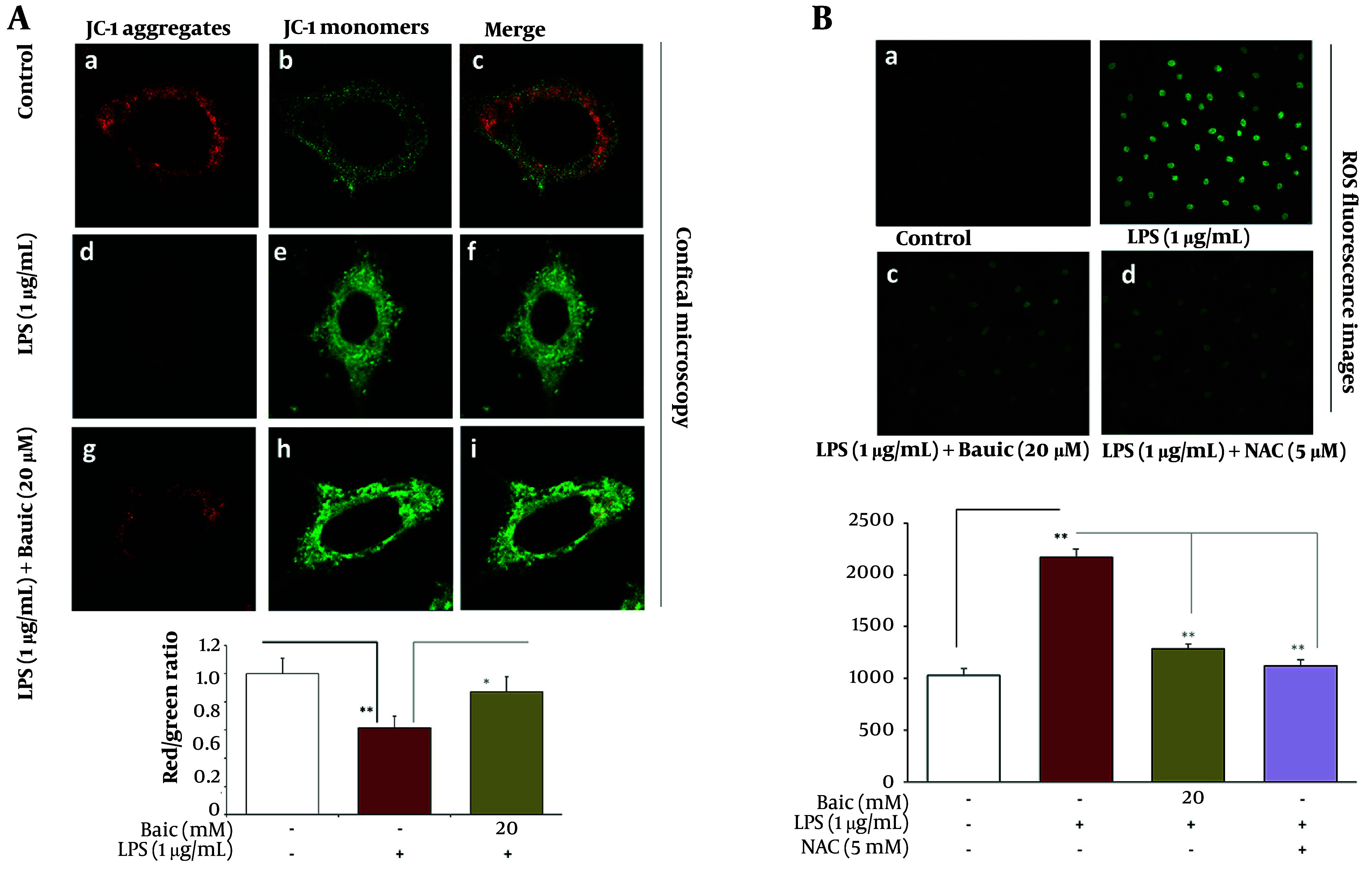
Baicalein preserves mitochondrial membrane potential (ΔΨm) and attenuates ROS overproduction during lipopolysaccharide (LPS)-induced mitochondrial stress. A, JC-1 mitochondrial membrane potential assay. Cells were stained with JC-1 (ab113850; 10 µg/mL, 20 min, 37°C) and imaged using a Leica TCS SP8 confocal microscope with a 60× oil objective. Red J-aggregates (polarized mitochondria) and green monomers (depolarized mitochondria) were quantified using ImageJ. ΔΨm was expressed as the red/green ratio. B, DCFH-DA ROS quantification. Cells were incubated with 10 µM DCFH-DA (30 min, 37°C), washed, and imaged by Leica SP8 confocal microscopy (40×). Fluorescence intensity was normalized to cell number. N-acetyl-L-cysteine (NAC) (5 mM) served as the antioxidant control.

To determine whether the protective effects of baicalein involve mitochondrial preservation, the mitochondrial membrane potential (ΔΨm) was assessed using the JC-1 fluorescence ratio (red/green), a sensitive indicator of mitochondrial polarization (Figure 4A). LPS exposure caused marked mitochondrial depolarization, as reflected by a sharp decline in the JC-1 ratio (P < 0.001), confirming mitochondrial dysfunction during inflammatory stress. Baicalein pretreatment mitigated this loss and achieved near-complete recovery (P < 0.01 vs LPS).

To further define the oxidative component of LPS-induced mitochondrial injury, intracellular ROS levels were quantified using the DCFH-DA fluorescence assay (Figure 4B). Consistent with the depolarization observed in Figure 4A, LPS markedly elevated total ROS, reaching approximately 220% of control (P < 0.001), confirming robust oxidative stress. Baicalein pretreatment significantly attenuated ROS. The high-dose baicalein group exhibited a stronger reduction, lowering ROS to 125% of control (P < 0.001 vs LPS). To validate that mitochondrial protection was ROS-dependent, a reference antioxidant control (LPS + NAC) was included (Figure 4B, panel d). NAC reduced ROS to 150% of control, a range closely comparable to high-dose baicalein. The similarity between LPS + NAC and LPS + Baic-High confirms that baicalein’s mitochondrial stabilization occurs primarily through suppression of ROS overproduction, rather than through unrelated signaling pathways. Together, these findings demonstrate that baicalein effectively limits oxidative stress, and its antioxidative action mirrors that of NAC, supporting ROS suppression as a core mechanism underlying its mitochondrial and cytoprotective effects.

## 5. Discussion

**Figure 5.**
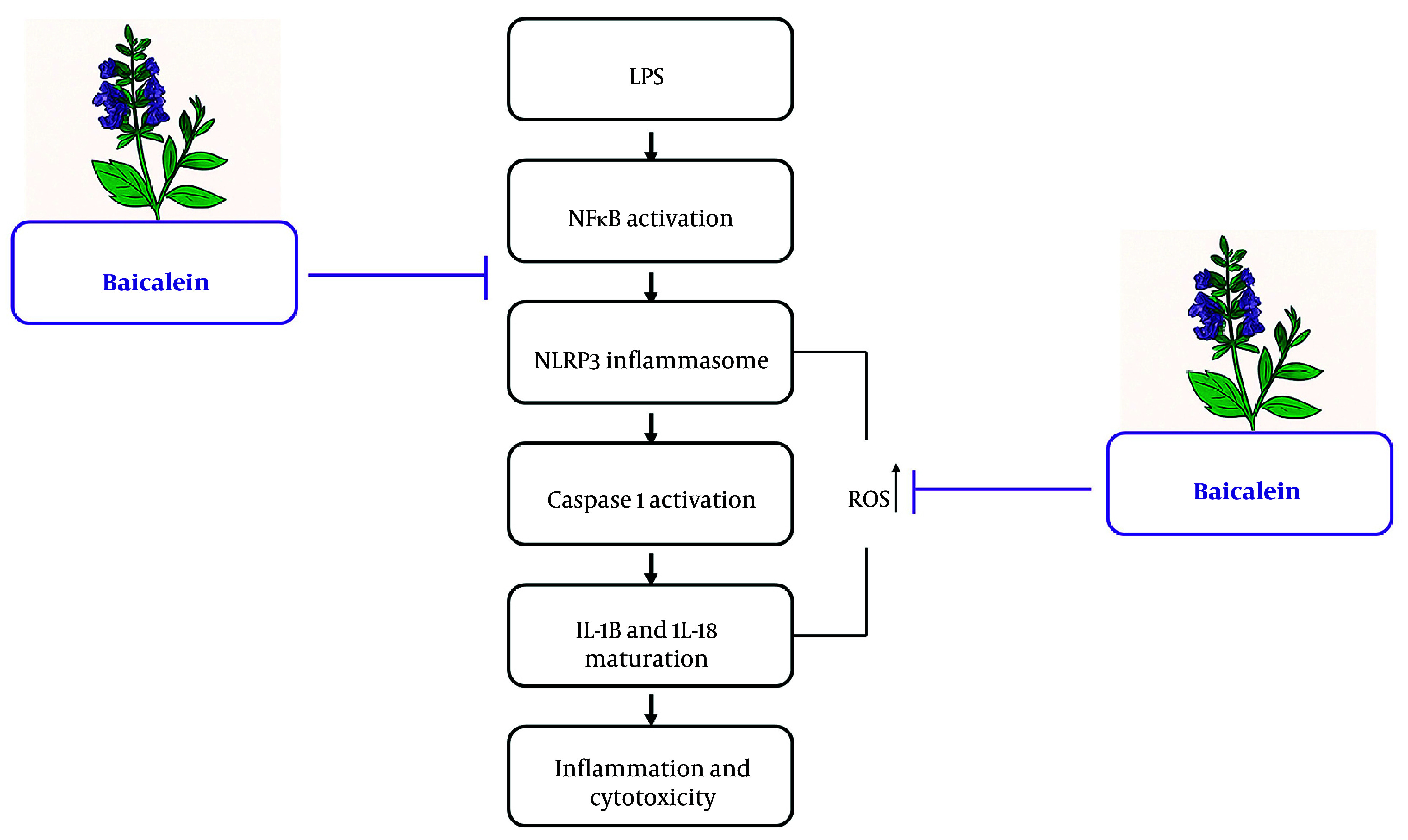
Proposed mechanistic model depicting the inhibitory effects of baicalein on lipopolysaccharide (LPS)-induced inflammasome activation in H9c2 cardiomyoblasts. Baicalein exerts multilevel suppression of the LPS-triggered inflammatory cascade in H9c2 cardiomyoblasts. Following LPS exposure, canonical TLR4 signaling activates NF-κB, leading to transcriptional priming of NLRP3, Casp1, and Il1b. Mitochondrial stress and elevated ROS further promote NLRP3 inflammasome assembly, resulting in caspase-1 activation and proteolytic maturation of IL-1β and IL-18, culminating in inflammatory cytokine release and cytotoxic injury. Baicalein, administered prior to LPS challenge, inhibits both the priming arm (NF-κB activation and NLRP3 induction) and the activation arm (mitochondrial dysfunction, ROS overproduction, and caspase-1 activity), thereby limiting downstream IL-1β/IL-18 maturation and reducing inflammation-associated cytotoxicity. The schematic summarizes the integrated points of interference experimentally demonstrated across Figure 1 - 4.

This study makes it clear that baicalein produces strong anti-inflammatory and antioxidative protective effects against LPS-induced damage in H9c2 cardiomyoblasts through the concurrent inhibition of NF-κB activation, the NLRP3 inflammasome, caspase-1 activity, IL-1β/IL-18 secretion, and mitochondrial dysfunction. Taken together, the current observations place baicalein at the top of the list for its use as a modulator of sepsis-induced cardiomyopathy related to the inflammation/mitochondria pathway. This study is very useful in providing the sequence of inflammasome priming, cytokine biogenesis, and mitochondrial disruption due to LPS stimulation. In the mechanistic summary presented in Figure 5, the anti-inflammatory activity of baicalein is emphasized as targeting various inflammatory milestones within the LPS-NF-κB-NLRP3 cascade in H9c2 cardiomyoblast cells.

Lipopolysaccharide activation quickly translates into NF-κB activation to initiate the transcriptional priming of NLRP3 and pro-IL-1β, followed by activation of the NLRP3 inflammasome, caspase-1 activation, and the subsequent processing of pro-IL-1β and IL-18 to their mature forms, resulting in inflammatory cytotoxicity. Baicalein modulates its anti-inflammatory activity by specifically acting on two key milestones of the LPS-NF-κB-NLRP3 cascade: (1) The first milestone is the NF-κB-mediated transcriptional activation step, inhibiting the expression of NLRP3 and pro-IL-1β, and (2) the second is the downstream effector level, inhibiting inflammasome formation, caspase-1 activation, and the eventual processing of mature IL-1β and IL-18. These dual actions of baicalein are consistent with our experimental evidence of reduced NLRP3 accumulation, decreased caspase-1 activity, reduced IL-1β/IL-18 release.

Early transcriptional kinetics in our study indicated that Nlrp3 and Il1b were maximally expressed at 2 hours after LPS stimulation, while Il18 showed very little transcriptional induction. These kinetic events are in keeping with the canonical two-signal mechanisms involved in NLRP3 activation, in which NF-κB priming is identified as the initial signal required for Nlrp3 and Il1b induction (30, 31). Also consistent with other reports is the minimal induction in Il18 mRNA levels. It has been suggested in earlier reports that Il18 is expressed in a more constant manner and is regulated mainly in terms of caspase-1 proteolytic processing (32). Baicalein pretreatment significantly reduced the transcriptional surge in Nlrp3 and Il1b in a concentration-dependent manner. This suggests interference with the NF-κB priming process involved in inflammasome activation. Consistent with this are data in peripheral macrophages and vascular cells demonstrating reduced baicalein concentration in p65 phosphorylation along with decreases in upstream IκBα (33).

On the protein level, LPS caused a profound increase in NLRP3 protein assembly, caspase-1 p20 activation, and mature IL-1β p17 processing, typical of canonical pathway activity. These LPS-primed increases were also abrogated by baicalein, suggesting effective intervention in both the priming and effector phases. Inhibition of NLRP3 assembly in non-cardiac contexts such as hepatic macrophages, renal epithelial cells, and microglia has been previously demonstrated with baicalein (34-38). However, these data also add credence to the existence of a potential pleiotropic effect of baicalein, one that influences inflammasome activation even in cardiomyoblast cells, a lineage characteristically vulnerable to mitochondrial oxidative damage. Moreover, dual-site inhibition, encompassing transcriptional priming and trans-inflammasome assembly, can be inferred and is consonant with baicalein's previously established ability to maintain a stable mitochondrial redox state. The functional output of inflammasome signaling was further confirmed by IL-1β and IL-18 secretion, both of which were dramatically elevated after 24 hours of LPS stimulation.

Baicalein significantly suppressed the release of both cytokines in a dose-dependent manner. This is particularly noteworthy for IL-18, which is strongly implicated in sepsis-associated myocardial dysfunction and has been shown to contribute to contractile impairment via induction of nitric oxide synthase and calcium-handling abnormalities (39). The reduction in IL-18 secretion is consistent with the marked inhibition of caspase-1 enzymatic activity observed in our assay. Caspase-1 activity decreased from nearly ten-fold above control levels in LPS-treated cells to just above baseline in response to high-dose baicalein, confirming that baicalein interferes with inflammasome maturation at a critical enzymatic step. This finding aligns with the literature showing that ROS suppression can effectively limit caspase-1 activation by preventing the mitochondrial distress signals required for inflammasome assembly (40).

The mitochondrial data further support this integrated mechanism. Lipopolysaccharide induced pronounced mitochondrial depolarization, as seen by the reduced JC-1 red/green ratio, and caused a > 2-fold increase in intracellular ROS. This is consistent with studies showing that LPS disrupts electron transport chain function in cardiomyocytes, leading to massive ROS leakage that activates redox-sensitive inflammatory pathways (41). Baicalein significantly restored ΔΨm and reduced ROS levels in a dose-dependent manner. The antioxidant effect of baicalein was comparable to N-acetyl-L-cysteine (NAC), a classical ROS scavenger, reinforcing that the cardioprotective action of baicalein is at least partly mediated through redox regulation. Similar observations have also shown that baicalein protects cardiomyocytes from oxidative injury by enhancing mitochondrial biogenesis and inhibiting JNK signaling (42).

Our findings extend this work by linking mitochondrial ROS suppression directly to downstream inhibition of NLRP3 activation and caspase-1–dependent cytokine maturation. Importantly, the cytoprotective effects of baicalein were also reflected in MTT and LDH assays. LPS-induced metabolic inhibition and membrane damage were markedly attenuated by baicalein, consistent with previous reports that baicalein improves cell viability under oxidative and inflammatory stress (43, 44). The preserved spindle-shaped morphology in baicalein-treated cells further indicates structural protection, which may be attributed to stabilized cytoskeletal and mitochondrial networks. This reinforces conclusions from resveratrol and quercetin studies, in which antioxidant flavonoids mitigated contractile dysfunction in H9c2 cells exposed to endotoxin or hypoxic stress (45-47).

In addition to the mechanistic literature directly supporting the TLR4/NF-κB/NLRP3/ROS cascade, broader evidence further reinforces the biological plausibility and translational relevance of targeting redox–inflammation coupling with polyphenol/flavonoid-like interventions. Recent studies have linked antioxidant capacity with downstream inflammatory readouts across experimental settings, supporting the concept that improving cellular redox balance can attenuate inflammation-driven tissue injury and preserve cellular function, while also emphasizing the importance of dietary/plant-derived bioactives and health-behavior determinants in cardiometabolic risk contexts (48-53). Collectively, these reports align with our observation that baicalein’s suppression of ROS-associated signaling coincides with dampened inflammatory output and cytoprotection in cardiomyoblasts.

Taken together, our results position baicalein as a multitarget cardioprotective agent capable of attenuating several interconnected pathological pathways within the LPS–TLR4–ROS–NLRP3–caspase-1 axis. By inhibiting NF-κB activation, blunting NLRP3 priming, suppressing caspase-1 activation, restoring mitochondrial polarization, and reducing intracellular ROS, baicalein interrupts the positive feedback loop that drives inflammatory cardiomyocyte injury. This integrative mechanism corresponds well with the emerging concept that effects on mitochondrial health and redox balance have profound consequences for inflammasome regulation. Few studies have simultaneously assessed these multiple layers of regulation in a unified cardiac model, making the current investigation a valuable contribution to the literature.

Despite these strengths, some limitations should be acknowledged. H9c2 cells, while widely used, do not fully recapitulate the phenotype of adult ventricular cardiomyocytes, particularly in terms of contractile protein expression and metabolic maturity. Additionally, our study focused on canonical NLRP3 signaling, but other inflammasomes or non-canonical pathways (e.g., caspase-11 activation) may also contribute to LPS-induced cardiac injury. Future work employing primary cardiomyocytes, in vivo validation, or siRNA-mediated knockdown of NLRP3 and caspase-1 could further clarify the contribution of specific nodes within this pathway.

Nonetheless, the consistency among transcriptional, protein-level, enzymatic, mitochondrial, and cytokine data provides compelling evidence for baicalein’s therapeutic potential. In summary, this study demonstrates that baicalein provides significant protection against LPS-induced inflammatory cardiomyocyte injury by concurrently suppressing key steps along the sequential TLR4/NF-κB/NLRP3/mitochondrial ROS axis, including NF-κB priming, inflammasome activation, caspase-1 activity, cytokine maturation, and oxidative mitochondrial dysfunction. These findings advance our understanding of baicalein’s pharmacological actions in cardiomyoblasts and highlight the therapeutic relevance of targeting the ROS–inflammasome signaling cascade in sepsis-associated cardiomyopathy and related inflammation-driven cardiac disorders.

ijpr-25-1-169689-s001.pdf

## Data Availability

The dataset presented in the study is available on request from the corresponding author during submission or after publication.
