# Supplementary material for: Baicalein Protects H9c2 Cardiomyoblasts Against LPS-Induced Inflammatory Injury by Modulating the NF-κB/NLRP3 Inflammasome Pathway and Mitochondrial ROS
Source: Iran J Pharm Res. 2026 Apr 28;25(1):e169689. doi: 10.5812/ijpr-169689 (PMC13187689; doi:10.5812/ijpr-169689)
Supplement: ijpr-25-1-169689-s001.pdf [file ijpr-25-1-169689-s001.pdf]

Supplementary Table S1. Primer sequences used for RT–qPCR

Gene	Forward primer (5'→3')	Reverse primer (5'→3')
**Nlrp3**	CAGCGATCAACAGGCGAGAC	AGAGATATCCCAGCAAACCTATCCA
**Il1b**	CCCTGAACTCAACTGTGAAATAGCA	CCCAAGTCAAGGGCTTGGAA
**Il18**	GACTGGCTGTGACCCTATCTGTGA	TTGTGTCCTGGCACACGTTTC
**Casp1**	ACTCGTACACGTCTTGCCCTCA	CTGGGCAGGCAGCAAATTC
**GAPDH**	GAACATCATCCCTGCATCCA	CCAGTGAGCTTCCCGTTCA
